# Opium in Nervous Fevers, Etc.

**Published:** 1855-12

**Authors:** Morton Dowler

**Affiliations:** New Orleans


Opium in Nervous Fevers, etc.—*Typhus.* (Revue de Thérapeutique Médico-Chirurgicale of June 15, 1855.) By M. Morton Dowler, M. D., of New Orleans.

We meet, sometimes, among subjects who are very nervous and irritable, certain fevers without any very determined character. These are fevers which proceed from veneral, oanic, or alcoholic excesses, (*delirium tremens febrile*,) or from fatigues, from watchings, or from study, etc. In this state of nervous erethism, we employ, with great advantage, some light, opiate preparation, with a demulcent restorative, and lightly analeptic regimen. In this manner we remove the febrile erethism, by removing the over-excited nervous system. The slow, hectic essential fever, (Broussais himself has proven its essentiality,) may be treated in the same manner. In relation to the fever of convalescents, if there be appetite, the best remedy is a light restorative and alimentary regimen, studiously adapted to the condition of the digestive organs, and not the useless, not to say suicidal, excess in diet. One thing is certain, convalescents do not disembarrass themselves of their fever without moderation in eating, as we have often observed.

In relation to typhous, though from the period of its invasion in Paris we have observed a great number of cases, we ought to here state that we have never seen opium exhibted in this terrible disease. For this reason, in order to fill this hiatus, we shall here cite a remarkable passage from Hufeland. This illustrious practioner holds the following language in relation to the treatment of typhus:

"Opium is salutary, when, after having sufficiently practiced blood-letting, cold applications, and evacuents, the signs of congestion disappear, though the delirium persists, or should it ever run into furor. The condition, then, is purely nervous, and in numerous cases we obtain the entire favorable effect of opium, though it is better to combine it with calomel. I shall never forget the joy it brought me in the case of one of my cherished colleagues. The patient was in the seventh day of a most intense typhus, pulse small and so frequent that it could scarcely be counted, accompanied with coma, delirium, and subsultus tendinum. Blood-letting, cold purgatives, calomel, all had been largely employed. I gave him the powders of calomel and opium, before mentioned, and at the sixth dose the pulse became stronger and slower, the spasms had ceased, the head was clearer, and a crisis immediately appeared. The amelioration began from this time, and went forward regularly. How many other cases I might here cite of an analogous character!

"Opium is further salutary when the typhus is accompanied from the beginning by diarrhœa, or dysentery, or cholera, acting as a powerful derivative in relation to the brain, but with danger of total exhaustion of its forces and of death from inanition. Opium is our only means of arresting these profluviæ, of calming this super-excitation of the intestinal canal, and of saving the life of the patient. We must, however, take care that the primæ viæ be first freely cleared. Opium was the only agent that proved efficient in the typhus which ravaged Prussia 1806–7, of which diarrhœa was the essential accompaniment."—*Manual of Practical Medicine*, p. 727.

For contagious typhus, see Pringle, who has accurately described it, and especially consult the excellent work of Hidelbrand on Typhus, in which will be found excellent rules for the employment of calmatives in the nervous period of the disease.

With these general considerations, which we submit to the consideration of the profession, we take leave of the subject. We have not brought into view either the plegmasia, or all the other diseases in which opium may be happily applied. This would require a volume, and we have no inclination to produce a complete opiology.

We conclude here, by reproducing what we have said on the subject of opium in our *Thérapeutique Appliquée*, fourth edition:

"We are convinced that without opium there would literally be no therapeutic means in relation to painful chronic disease. Were we deprived of this magic remedy, of this soothing and beneficent agent, we should consider ourselves without resource, and would at once renounce the practice of medicine. Sydenham gave thanks to God for having bestowed opium on man to cure the innumerable diseaes which weigh him down and take away his life."

Meanwhile, it is with opium as with all our heroic remedies—it is a two-edged sword, and can do much good and much evil, accordingly as it is well or ill applied.

Sacra vitæ anchora, circumspecte agentibus
Est opium, cymba Charontus in manu imperiti.—Wedel.

[*Stethoscope.*

